# Prolonged activity of HERV-K(HML2) in Old World Monkeys accounts for recent integrations and novel recombinant variants

**DOI:** 10.3389/fmicb.2022.1040792

**Published:** 2022-12-01

**Authors:** Saili Chabukswar, Nicole Grandi, Enzo Tramontano

**Affiliations:** Laboratory of Molecular Virology, Department of Life and Environmental Sciences, University of Cagliari, Cagliari, Italy

**Keywords:** HERV-K, HML2, Old World Monkeys, Macaca, recombination, HERV-K11, viral evolution, endogenous retroviruses

## Abstract

Around 8% of the human genome comprises Human Endogenous Retroviruses (HERVs) acquired over primate evolution. Some are specific to primates such as HERV-K, consisting of 10 HML subtypes and including the most recently acquired elements. Particularly, HML2 is the youngest clade, having some human-specific integrations, and while it has been widely described in humans its presence and distribution in non-human primates remain poorly characterized. To investigate HML2 distribution in non-human primates, the present study focused on the characterization of HML2 integrations in *Macaca fascicularis* and *Macaca mulatta* which are the most evolutionarily distant species related to humans in the *Catarrhini* parvorder. We identified overall 208 HML2 proviruses for *M. fascicularis* (77) and *M. mulatta* (131). Among them, 46 proviruses are shared by the two species while the others are species specific. Only 12 proviruses were shared with humans, confirming that the major wave of HML2 diffusion in humans occurred after macaques’ divergence. Phylogenetic analysis confirmed structural variations between HML2 macaques’ species-specific proviruses, and the ones shared between macaques and humans. The HML2 loci were characterized in terms of structure, focusing on potential residual open reading frames (ORFs) for *gag, pol,* and *env* genes for the latter being reported to be expressed in human pathological conditions. The analysis identified highly conserved *gag* and *pol* genes, while the *env* genes had a very divergent nature. Of the 208 HML2 proviral sequences present in Macaca species, 81 sequences form a cluster having a MER11A, a characteristic HML8 LTR sequence, insertion in the env region indicating a recombination event that occurred between the HML2 *env* gene and the HML8 LTR. This recombination event, which was shown to be present only in a subset of macaques’ shared sequences and species-specific sequences, highlights a recent viral activity leading to the emergence of an *env* variant specific to the Old World Monkeys (OWMs). We performed an exhaustive analysis of HML2 in two species of OWMs, in terms of its evolutionary history, structural features, and potential residual coding capacity highlighting recent activity of HML2 in macaques that occurred after its split from the *Catarrhini* parvorder, leading to the emergence of viral variants, hence providing a better understanding of the endogenization and diffusion of HML2 along primate evolution.

## Introduction

Human Endogenous Retroviruses (HERVs) are the retroviral portion of our DNA: they have been acquired from the ancient integration of extinct exogenous retroviruses and account for up to 8% of the human genome (Blikstad et al., 2008; Escalera-Zamudio and Greenwood, 2016). The integration of these retroviral elements is the result of the vertical transmission of the proviruses that are integrated in germline cells through a Mendelian heritability fashion (Garcia-Montojo et al., 2018). A typical HERV structure includes major internal portions of *gag, pro, pol,* and *env* genes flanked by long terminal repeats (LTRs) on both ends. While LTRs are needed for proviral integration and have mainly a transcriptional regulatory function, the four internal regions encode for viral enzymes and structural proteins. In particular, *gag* contains capsid (CA), matrix (MA), and nucleocapsid (NC) domains; *pro* encodes for viral protease (PR); *pol* includes reverse transcriptase (RT), ribonuclease H (RH), and integrase (IN) enzymes; and *env* is made of surface unit (SU) and transmembrane (TM) unit domains (Boller et al., 2008; Vargiu et al., 2016; Pisano et al., 2019). HERVs have been classified into three classes, i.e., Class I (gamma- and epsilon-like), Class II (beta-like), and Class III (spuma-like). Class II (beta-like) is the HERVK supergroup, which includes 10 Human-MMTV-like groups (HML1-10). Out of the 10 members, HML2 is the most active and recent member of the supergroup (Hohn et al., 2013).

The HML2 member of the HERVK family constitutes around 1% of the classified HERV classes and has been active and infectious for about 30 million years. The group has been well characterized by Subramanian et al. (2011) identifying 89 HML2 loci. Like the other classified HERVs, the HML2 proviruses are the results of insertion, deletion, and recombinational events making them potentially pathogenic (Grandi and Tramontano, 2017; Grandi et al., 2019; Pisano et al., 2021); they include four genes (*gag, pro, pol,* and *env*). The HML2 integrations are subdivided into two proviral subtypes (type I and type II; Xue et al., 2020) due to the presence of alternative splicing variants of *env*, producing Np9 (type I) and Rec (type II) proteins, differing for the presence or absence of the 292 bp deletion in the *env* gene, respectively (Acton et al., 2019). The Rec protein functions similarly to the Rev protein of human immunodeficiency virus (HIV) and Rex protein of human T cell leukemia virus (HTLV). These accessory proteins bind to viral transcripts and facilitate the viral genome incorporation in the cell cytoplasm (Pisano et al., 2021). On the contrary, Np9 is expressed in various cancers and is speculated to activate certain oncological pathways, but its function in HML2 is still not known (Zare et al., 2017; Liu et al., 2020).

A major focus of HERVs investigation is their expression in various physiological and pathological conditions that might provide potential functional roles of these insertions. Accordingly, HML2 expression has been studied in various cancer types such as lung cancer, germ cell tumors, prostate cancer, melanoma, etc. (Wang-Johanning et al., 2007; Chang et al., 2019; Salavatiha et al., 2020; Gao et al., 2021). Mechanisms studied for the upregulation of HML2 in carcinogenesis include chromosomal rearrangements, LTR-induced upregulation of oncogenes, HML2-derived oncoproteins, and HML2-mediated immunosuppression (Salavatiha et al., 2020). Apart from cancer, the expression of HML2 in neurological disorders such as ALS, Parkinson’s disease, and Alzheimer’s disease (Christensen, 2016; Douville and Nath, 2017; Küry et al., 2018; Dembny et al., 2020; Giménez-Orenga and Oltra, 2021; Kristensen and Christensen, 2021). Diabetes and other autoimmune diseases have also been a topic of research (Balada et al., 2010; Krzysztalowska-Wawrzyniak et al., 2011; Gröger and Cynis, 2018; Kremer et al., 2020). Even though these integrations have provided some insights on the expression patterns of HML2, the functional consequences of such expression patterns in macaques are still unclear. Hence, the present study can be the starting point for analyzing such expression patterns in detail.

As mentioned above, the HML2 group has been well characterized in our genome and compared to the other *Hominidae* which includes Gibbon, Orangutan, Gorilla, and Chimpanzee belonging to the *Catarrhini* parvorder and thus reporting 89 HML proviruses in the human genome by Subramanian et al. (2011), plus an additional HML2 element in chromosome 6q11.1 that was identified in the present study. Despite subsequent studies suggesting a recent activity and possible impact of HML2 group in humans, its presence and activity in OWMs has been less explored and it remains still unclear. In fact, while HERV characterization in the human genome gained considerable attention in the last decades, very few studies were dedicated in turn to the same analysis in non-human primates, including HERVK (HML7 and HML10) and HERVW. Overall, these studies suggested the accumulation of these integrations in the non-human primates as well as, based on the ancient circulation of a common viral ancestor that infected multiple primate lineages along their speciation (Grandi et al., 2016, 2017, 2018, 2020, 2021). The emergence of the human HML2 type I (Np9) has been proposed to take place after the split of OWMs from the other *Catarrhini* primates and hence, the macaques should only have the type II (Rec) HML2 proviruses (Heyne et al., 2015). Among the few studies investigating ERV in macaques, a study was specifically focused on identifying the presence of HML2 in Rhesus macaque and identified 19 HML2 proviruses, designating them as RhERV-K (Romano et al., 2007), while other studies focused on the envelope expression of HML2 in rhesus species (Romano et al., 2007). However, a thorough understanding of HML2 presence in OWM is still lacking. Thus, our study aimed to the depth characterization of the presence, evolutionary history, structure, and coding capacity of HML2 in OWMs, specifically in *Macaca fascicularis* (macfas) and *Macaca mulatta* (rhesus). Our search in the macaques’ genomes was performed to identify the HML2 integrations shared with humans as well as the ones present specifically in one or both OWM species, leading to the identification of 77 HML2 in macfas and 131 in rhesus, exhaustively depicting the group diffusion in these species. Particularly, we recognized a major group of HML2 integrations characteristics of both Macaca species, highlighting a recent activity of a different HML2 proviral subtype circulating in OWMs after their split from humans. Finally, for the first time, we identified the presence of recurrent secondary insertions in the HML2 *env* gene of macaques that might have been derived through a massive recombinational event which occurred between *env* gene of HML2 and the LTR region of HML8. Such an event has not been detected in any of the human HML2 sequences, revealing how it is specifically linked to the group diffusion in OWMs.

## Materials and methods

### Identification of HML2 integrations in human and macaques genomes

The identification of HML2 elements in *M. fascicularis* and *M. mulatta* genome assemblies was performed with a double approach, including comparative genomics with respect to humans and *de novo* BLAT searches.

#### Comparative genomics

With respect to humans, we started with refining the HML2 genomic coordinates reported in Subramanian et al. (2011) by searching them in GRCh38/hg38 genome assembly in the UCSC Genome Browser, after their conversion from the originally reported GRCh37/hg19. Once identified, the coordinates of each sequence were manually checked and refined in case of either additional or missing portions. In addition, for each HML2 element, we checked the primate species that acquired the common viral ancestor, i.e., the first species in which a specific HERV is found, being thus shared with more than one species that descended from a common primate ancestor. The validated human coordinates have been then used to move to the correspondent orthologous position in reference genome assemblies of green monkey, macfas, rhesus, baboon, and proboscis monkey (Chlorocebus_sabaeus 1.1/chlSab2, Macaca_fascicularis_5.0/macFas5, Mmul_10/rheMac10, Pani_3.0/papAnu4 and Charlie1.0/nasLar1, respectively), verifying the actual presence of the HML2 sequence. In both cases, the specific correspondence between human and OWM HML2 elements has been assured considering the univocal genomic flanking sequences. We further narrowed down the comprehensive HML2 identification in macfas and rhesus genomes.

#### *De novo* BLAT searches

To identify the HML2 elements having no orthologous sequences in humans, we performed a BLAT search in the above Macaques genome using Dfam HERV-K (DF0000188) as a query sequence in the UCSC Genome Browser. The hits obtained from the search were confirmed as HML2 by using the Repeat Masker annotations, excluding the ones already identified in the above comparative genomics approach. The obtained coordinates have been then compared to further distinguish the HML2 elements shared by the two Macaca species from the ones specific to one or the other.

After the final selection of the genomic coordinates, the latter have been used to build two BED files, one for each Macaca species, and to retrieve the corresponding nucleotide sequences from the respective genome assemblies.

### Sequence alignment and phylogenetic analysis

The HML2 sequences retrieved from human, Rhesus, and macFas genome assemblies were aligned all together as well as in dedicated alignments for various categories: (a) All sequences from the same species; (b) Sequences shared between both macaques but absent in humans; (c) Sequences of macfas and rhesus shared with humans; and (d) Species-specific sequences found only in macfas or rhesus. All the alignments were performed with Mafft algorithm G-INS-i and scoring matrix of 200PAM/k = 2 of Geneious Prime software (Biomatters Ltd., Auckland, New Zealand) using a proviral reference made with the Dfam HML2 internal portion (HERV-K) flanked by Dfam HML2 LTRs (LTR5). The reference sequence was annotated in the *gag* (~156–2,297)*, pro* (~2063–3,082)*, pol* (~3,037–5,785), and *env* (~5,624–7,951) regions to understand the variations such as insertions or deletions in the Macaque species.

Phylogenetic trees for the sequence alignments were built with MEGA11 software using the neighbor-joining (NJ) method and applying p-distance model. All the trees were tested by bootstrap method with 500 replicates. Likewise, to the alignment categories, trees were built for the complete proviral sequences of: (a) All HML2 sequences (human, macfas, and rhesus); (b) Sequences shared between Macaques, and (c) Species-specific sequences, to analyze the phylogeny of HML2 proviruses. Trees were also constructed for *gag* and *env* regions by extracting the corresponding gene sequences from the above alignments.

### Estimating the time of HML2 integrations

The age estimation for all the sequences of macfas and rhesus was based on the percentage of nucleotide divergence (D%) as obtained with multiple calculation approaches: (Escalera-Zamudio and Greenwood, 2016) LTR vs. LTR, i.e., 5′ and 3′ LTR of each proviral sequence; (Blikstad et al., 2008) single LTR vs. a consensus sequence generated from the alignment of all LTRs; (Garcia-Montojo et al., 2018) proviral *gag* and *pol* portions vs. the consensus generated from the alignment of each gene. For consensus-based approaches, a dedicated consensus sequence was generated for each supported cluster as observed in the corresponding phylogenetic tree. MEGA 11 was used for calculating the D% values for each category by applying the pairwise deletion option. The hypermutating CG dinucleotides were excluded for better divergent values. The time of integration (T) for all categories was obtained by the formula T = D%/0.34, in which 0.34 represents a rate of substitution in primates’ genome of 0.34%/nucleotide/million years. The same substitution rate has been used to estimate HERV T in previous studies (Subramanian et al., 2011). For LTR vs. LTR comparison, T was further divided by a factor of 2 considering that each LTR acquired mutations independently.

### Structural characterization and coding capacity

From the alignments performed as mentioned above, sequences of full-length or near full-length with respect to the reference sequence were selected. The structural characterization focused on identifying the potential residual *gag* and *env* ORFs (Open Reading Frames). All the insertions and deletions in the sequences were analyzed as compared to Dfam proviral reference, and their frequency was evaluated to have a clear understanding of the divergence in *gag* and *env* genes and to infer the possible presence of different variants of HML2.

The *gag* and *env* ORFs selected based on their structural completeness were translated in all forward frames using the Geneious software along with UniProt representative proteins for HML2 Gag (Q9HDB9, Q7LDI9, P87889, P63145, P63130, and P6312) and Env (Q69384, Q902F8, Q9UKH3, P61567, O71037, and O42043) to identify those with residual coding potential, i.e., without internal stop codons and frame shifts. The selected amino acid sequences were finally evaluated for the conservation of structural and functional domains using the above UniProt annotations and NCBI Conserved Domains tool. Recombination events between HML2 *env* gene and HML8 MER11A LTR were assessed through the software Recco (Maydt and Lengauer, 2006).

## Results

### Identification of HML2 integrations in human and macaques genomes

To identify the presence of HML2 in OWMs, we used two approaches: (Escalera-Zamudio and Greenwood, 2016) comparative genomics, to identify the HML2 orthologous to human integrations and (Blikstad et al., 2008) *de novo* identification, to retrieve HML2 elements not shared with humans. For comparative genomics, we first manually checked and refined the HML2 coordinates as reported previously (Subramanian et al., 2011) using the GRCh38/hg38 human genome assembly. As the human HML2 proviral sequences reported in Subramanian et al. (2011) were identified using a previous human genome assembly (GRCh37/hg19), we converted and checked one by one their coordinates in hg38 assembly. While refining the coordinates, using the Blast-like alignment tool (BLAT) we reported one new HML2 proviral sequence. Hence, according to the current human genome assembly, the human genome consists overall of 90 HML2 integrations: of these, 89 were described in Subramanian et al. (2011) and 1 additional proviral HML2 locus (6q11.1). Hence, overall, 90 human HML2 full-length and near full-length sequences were considered (Supplementary Table S1). The search of these 90 HML2 elements in the non-human primates (green monkey, macfas, rhesus, baboon, and proboscis monkey) genomes determined that only 8 complete proviral sequences were shared with the green monkey genome, 12 of them are shared with macfas and rhesus, and 11 and 7 of them with Baboon and Proboscis monkey, respectively (Supplementary Table S1; Supplementary Figure S1). As reported in materials and methods, for each HML2 element, we identified the common viral ancestor, i.e., the first proviral integration found in a certain primate species and thus shared by the other species that descended from the same common ancestor. Hence, its identification for the rest of elements not shared with the OWM genomes confirmed that they were acquired by primate lineages after the evolutionary divergence of macaques, being hence found only in *Hominidae* primates (gibbon, orangutan, gorilla, and chimpanzee; Supplementary Table S1).

For the *de novo* identification of additional HML2 integrations in OWM genomes, a BLAT search was conducted using Dfam HML2 reference sequence as a query to obtain a list of hits in Chlorocebus_sabaeus 1.1/chlSab2, Macaca_fascicularis_5.0/macFas5, Mmul_10/rheMac10, Pani_3.0/papAnu4, and Charlie1.0/nasLar1 genomes. All the coordinates were checked, and exact coordinates were used to extract the HML2 nucleotide sequences. With this approach, we identified a total of 81 HML2 proviruses in Green Monkey, 77 in Macfas, 131 HML2 integrations in Rhesus, 76 in Baboon, and 102 in Proboscis monkey (Table 1). Due to the poor annotations between the non-human primates and primate genomes, we narrowed our studies to macfas and rhesus as these are the most widely used models for biomedical research (Bunlungsup et al., 2017).

**Table 1:** HML2 integrations in OWM genomes.

	OWM genomes				
	Green monkey	Macfas	Rhesus	Baboon	Proboscis monkey
Human Shared HML2 loci	8	12	12	11	7
Total HML2 integrations^*^	81	77	131	76	102

* Including the above human-shared ones.

Further to understand the distribution of HML2 integrations in humans, macfas, and rhesus, we classified them into three categories: (a) Sequences of macfas and rhesus shared with humans (as identified in the above comparative genomics approach); (b) Sequences shared between both macaques but absent in humans; (c) Species-specific sequences found only in macfas or rhesus (Table 2). As shown in Table 2, besides the 12 HML2 elements common to humans (a), additional 46 HML2 loci are shared between the two Macaca species (b), of which one is found as a solitary LTR in macfas (Table 2; Supplementary Table S2). Finally, other 19 and 73 HML2 integrations were found specifically in macfas and rhesus genomes, respectively (c) (in blue in Supplementary Tables S3, S4, respectively).

**Table 2:** Distribution of HML2 loci in Humans, *Macaca fascicularis,* and *Macaca mulatta.*

	*Macaca mulatta*	*Macaca fascicularis*
**Comparative genomics of human-shared HML2**		
HML2 human loci (90)	12	12
***De novo* search for macaques’ specific HML2**		
Macaque-shared HML2	46	46
Macaque species specific	73	19
**Total HML2 integrations**		
	131	77

Overall, we identified a total of 208 HML2 integrations in macaques, of which 77 in macfas (12 shared with humans, 46 shared with rhesus, and 19 macfas specific) and 131 in rhesus (12 shared with humans, 46 shared with macfas, and 73 rhesus specific). Once identified, all the sequences were extracted in the FASTA format and aligned to the Dfam reference for HML2 group by dividing them into the above-mentioned categories to understand the similarities and differences among the sequences.

### Phylogenetic analysis of HML2 proviral sequences

**Figure 1:**
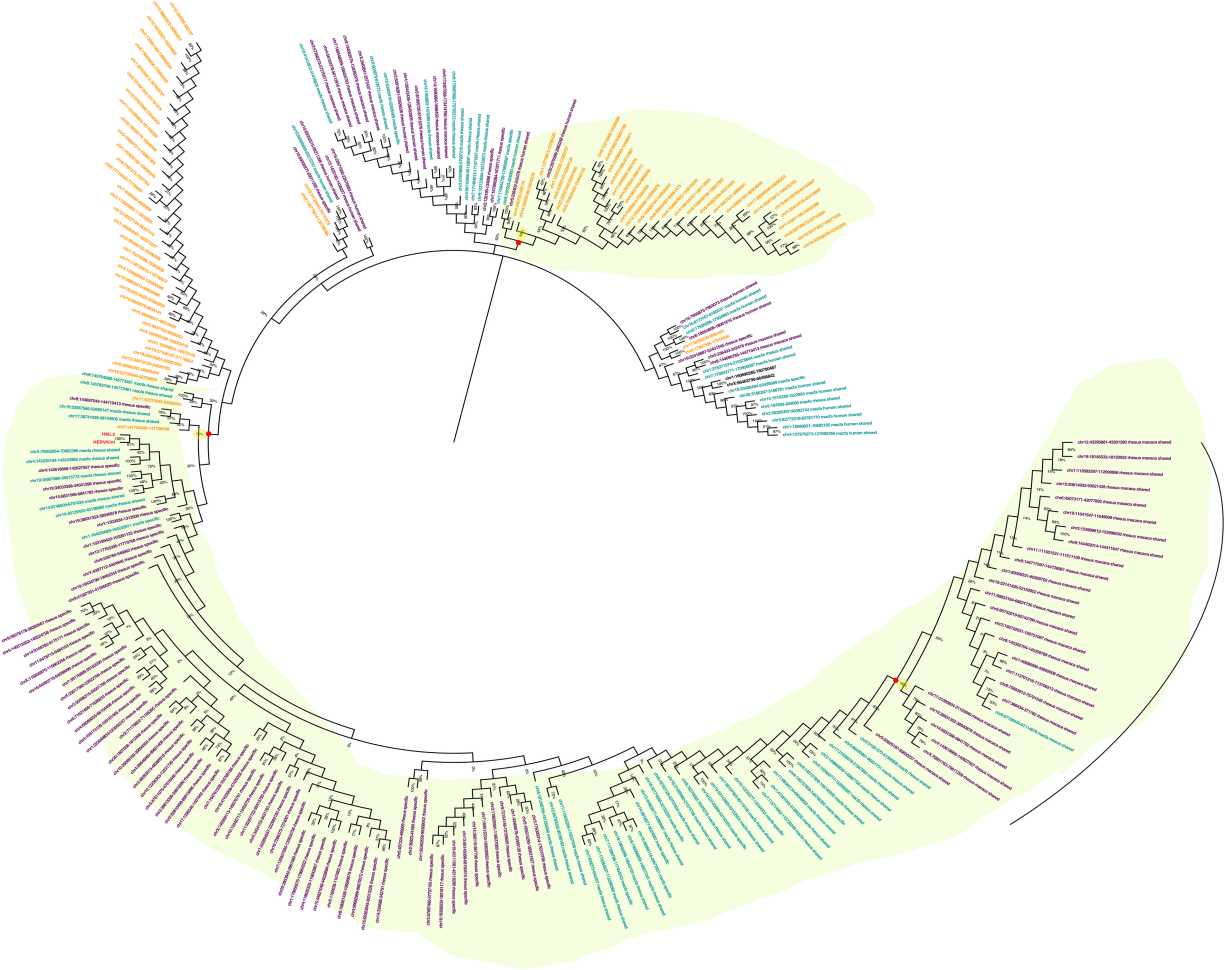
Phylogeny of HML2 integrations in Humans, *Macaca fascicularis* and *Macaca mulatta.* Phylogenetic trees generated for full-length or near full-length sequences for all the three species using Neighbor-joining method with a bootstrap of 500 replicates in MEGA 11 software. The human sequences are indicated in yellow, *M. fascicularis* in cyan, and *M. mulatta* in magenta. Supported phylogenetic clusters are highlighted with a light green shade.

**Figure 2:**
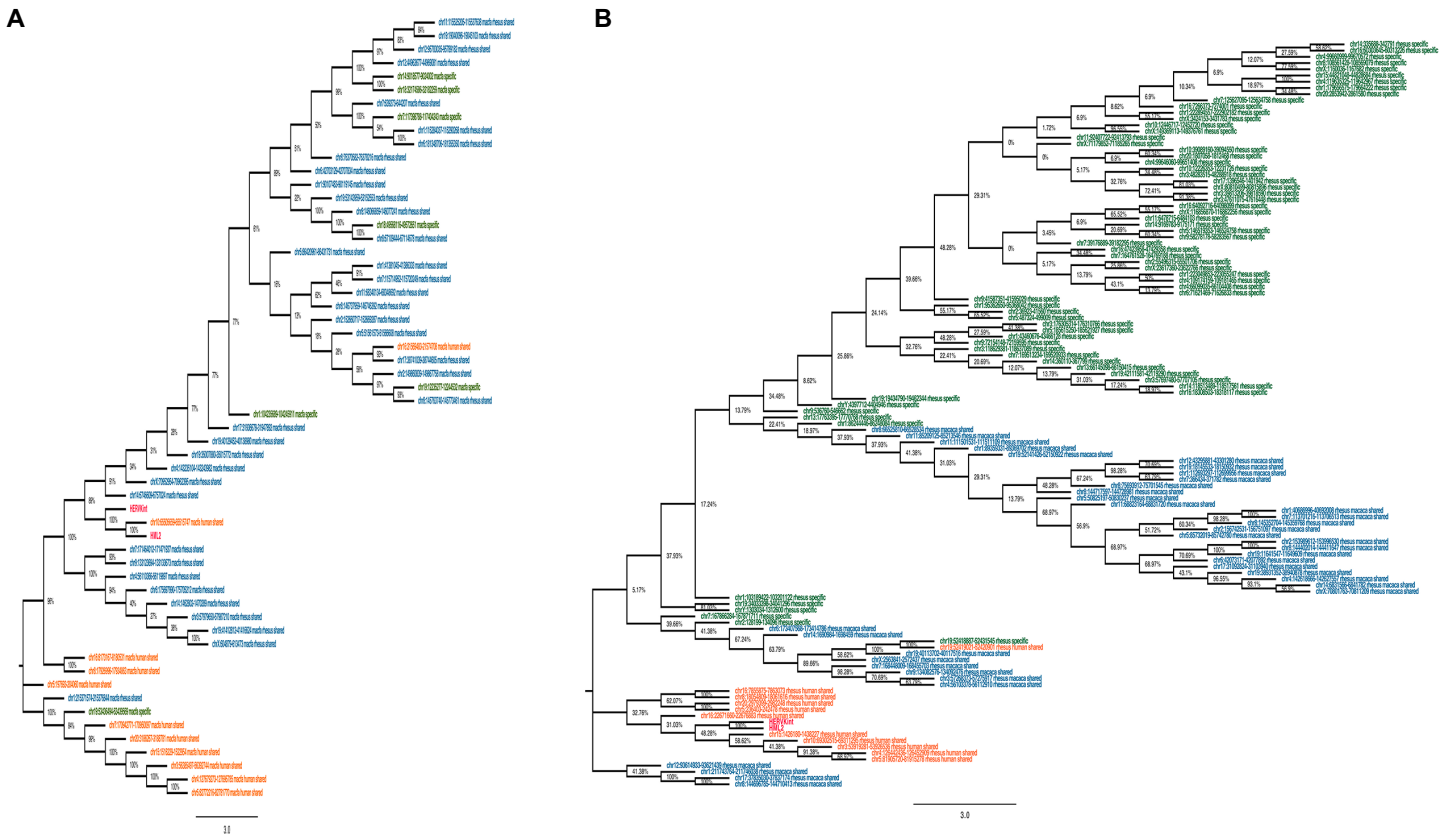
Phylogeny of HML2 loci in Old World Monkeys *Macaca fascicularis* and *Macaca mulatta*. Both trees represent the HML2 sequences that are specific to individual species (green), some common among the species (blue), and some shared with humans (orange). Lastly, the reference sequences are highlighted in red. Only the full-length sequences were included to generate the phylogeny by applying Neighbor-joining method with 500 bootstrap values in MEGA 11 software.

To understand the evolutionary history of HML2 acquisition by macaques, we infer their phylogeny by generating neighbor joining (NJ) trees with a bootstrap of 500 replicates for complete HML2 sequences (Figures 1, 2). To obtain reliable phylogenetic trees, only the full-length and near full-length sequences were included using Dfam HML2 prototype (HERVK) as a reference. First, we evaluated the overall phylogeny of the group, by including all the HML2 sequences retrieved from humans and the two macaques, to assess the major relationships among the three species (Figure 1). Observing the tree, it is clear that HML2 elements form clear clustering of macaque HML2 sequences and human HML2 without forming mixed grouping of HML2 from different species among the major clusters, while a minority of mixed grouping is observed in the lower part of the cluster having a lower bootstrap support. Looking at the phylogenetic clusters supported by bootstrap, the first one, shown in the upper part of the tree, groups HML2 sequences from the human genome, but also 2 macfas and 1 rhesus elements known to be shared with humans (bootstrap = 84%; Figure 1). The remaining human HML2 form a separate cluster, that showed low bootstrap support (54%), except for 4 human elements that grouped with OWMs sequences (Figure 2). The second-wide phylogenetic cluster includes the majority of OWMs HML2 elements and the HML2 group Dfam references, being supported by a bootstrap of 72% (Figure 1). We found in this cluster mostly HML2 sequences that were specific to one or the other Macaca species, plus those elements shared between the two but not present in humans (Figure 1). Even if these two classes of elements did not form specifically supported subclusters, they were grouped separately in this main cluster, except for a supported subcluster that includes 26 rhesus sequences shared with macfas out of 46 (bootstrap = 96%; Figure 1). Finally, a mixed group of sequences (bootstrap = 67%) was found at the root of the tree, including 2 human HML2 elements and some macfas and rhesus sequences (being either shared with humans or not; Figure 1).

Secondly, we evaluated HML2 phylogeny for the two individual Macaca species, building two NJ trees including all HML2 proviruses present in macfas (Figure 2A) and rhesus (Figure 2B). In these trees, sequences are colored based on the fact that they are shared with humans (orange), shared only between the two Macaca species (blue), or specific either to macfas or rhesus (green). As shown in the macfas NJ tree, HML2 elements form two major clusters, both supported by a bootstrap of 100% (Figure 2A). The lower cluster (8 sequences) includes HML2 elements shared with humans (orange, *n* = 6), while the upper cluster is formed exclusively by HML2 loci shared by the two macaques (blue) or specific to macfas (green). The upper cluster is further divided into two subclusters, also supported by high bootstrap values (100 and 89%), sharing a strong phylogenetic relation with two of the human-shared sequences as well (96%). The former subcluster comprises only HML2 elements shared by the two macaques (*n* = 8), while the second includes the group references and the majority of HML2 elements, corresponding to macaque-shared (*n* = 29) and mafias specific (*n* = 6) sequences plus 2 of the human-shared loci (Figure 2A). Particularly, macfas species-specific sequences did not form an individual cluster, but instead, they were distributed across the tree along with the macaque-shared ones. On the other hand, rhesus NJ tree did not show any major supported cluster, suggesting a homogeneous composition of the group (Figure 2B). However, also in this case, a clear grouping of (i) species-specific HML2 (green), (ii) sequences shared between the two macaques (blue), and finally (iii) those shared with humans (orange) was observed, still suggesting relevant differences among the three categories. More in detail, all HML2 sequences shared with humans formed a separate group including also the HML2 references, except for one element that was included in another cluster with macaque-shared proviruses (Figure 2B). A minority of HML2 macaque-shared and species-specific HML2 elements form small clusters in the lower part of the tree, while most are included in a big upper group, showing a dichotomy based on the category of belonging (Figure 2B).

### Time of integration

**Figure 3:**
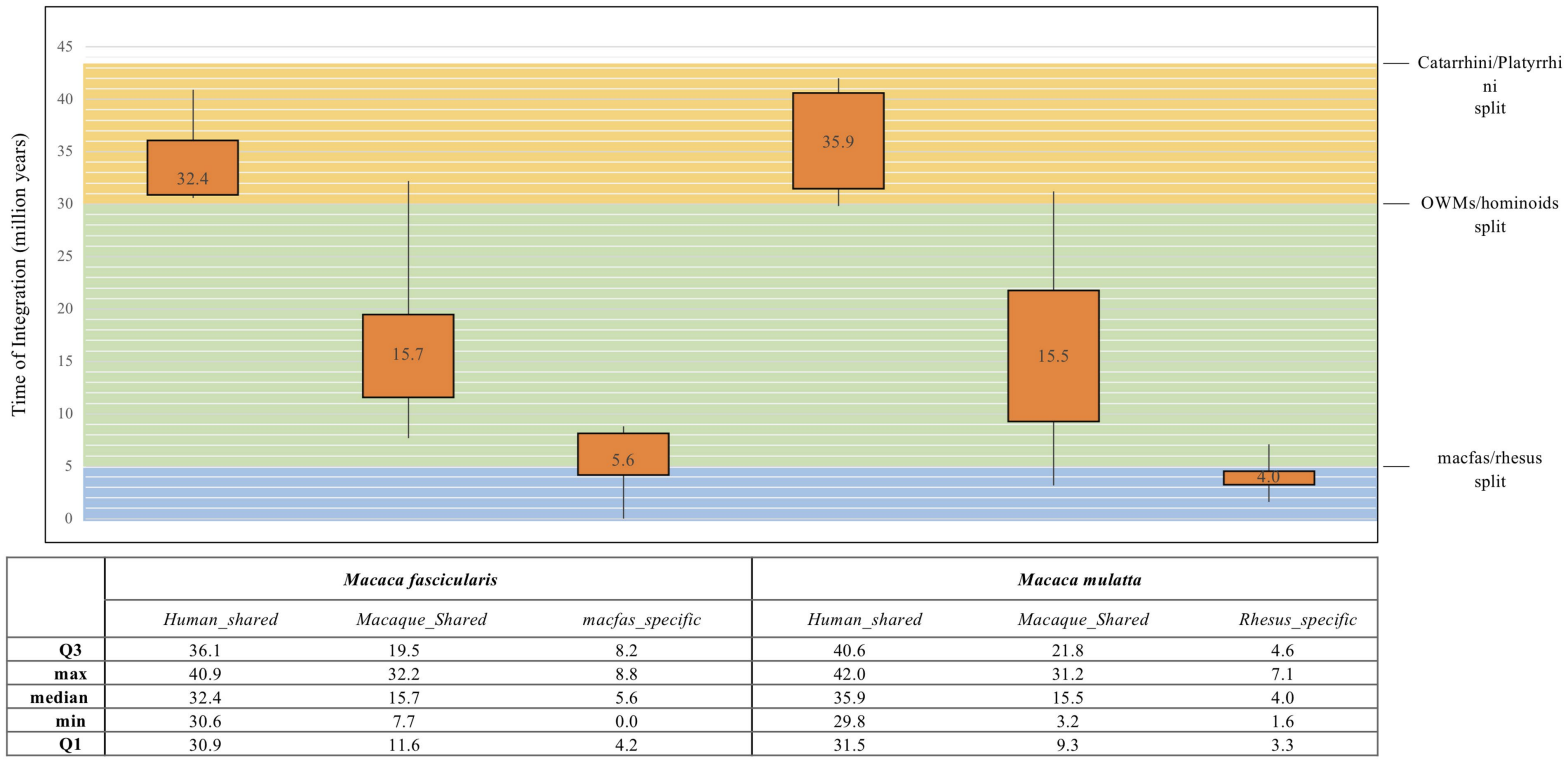
Box plot representation of HML2 diffusion in macaques. The age for all the three categories of macaque HML2 sequences (human shared, macaque shared, and species specific) were calculated by considering divergence between the (i) 5′ and 3′ LTR of same HML2 loci; (ii) LTR vs. Consensus generated; (iii) gag and pol genes against the consensus of each gene. The values in the box plot are the mean values. The theoretical age ranges for the three categories based on evolutionary divergence times are indicated with colored boxes for *Catarrhini/Platyrrhini* (around 43 mya, orange), OWMs/hominoids (around 30 mya, green), and macfas/rhesus (around 5 mya, blue).

The traditional mode of HERV age estimation relies on the fact that 5′ and 3′ LTRs of the same provirus are identical at time of integration and then accumulate mutations according to the host genome substitution rate. This allows to infer the time passed since their acquisition by calculating the nucleotide LTR divergence, obtaining the time of integration of each provirus. Even though this is a widely used method, it has some major limitations such as the exclusion of those HERVs that present only one LTR or lost both LTRs during the persistence in the host genome. Thus, we used a multiple approach of age estimation including (i) the above LTR vs. LTR divergence, combined with the consensus-based calculation of (ii) each LTR vs. consensus generated from LTR sequences, (iii) *gag* gene vs. *gag* consensus, and (vi) *pol* gene vs. *pol* consensus. In this case, the consensus, being generated from the alignment of all HML2 sequences, should somehow represent the ancestral HML2 sequence. This multiple approach was used for the three main categories of HML2 elements, i.e., human-shared HML2 loci, macaque-shared sequences, and species-specific sequences. The age estimates for the HML2 elements of each category were then used to build box plots to have a better picture of their dynamics of diffusion in macaques and in the *Catarrhini* parvorder (Figure 3). The age estimation of HML2 elements shared with humans in macfas (median age: 32.4 million years ago, mya) and rhesus (median age: 35.9 mya) indicates that these elements were acquired by primates around 34 and 30 mya, respectively. This is in line with their diffusion in human-macaques common ancestor until before the evolutionary split between macaques and *Hominidae* lineages (around 30 mya; Figure 3). Accordingly, the HML2 elements shared between the two macaques and not present in humans were acquired after such a split, with lower estimated median ages of 15.7 (macfas) and 15.5 (rhesus), but before the estimated split between the two Macaca species (around 5 mya). Lastly, species-specific HML2 integrations represented the youngest category, having been acquired after the separation of the two Macaca species. Particularly, macfas and rhesus species-specific insertions had a median value near to the estimated split (5.6 mya and 4 mya, respectively; Figure 3). The differences in these three categories based on the phylogenetic analysis and age estimation studies also hint the divergence in the structure of HML2 proviruses in humans, macfas, and rhesus, which are further explained in detail in the structural characterization below.

### Structural characterization of macaques’ HML2 provirus

**Figure 4:**
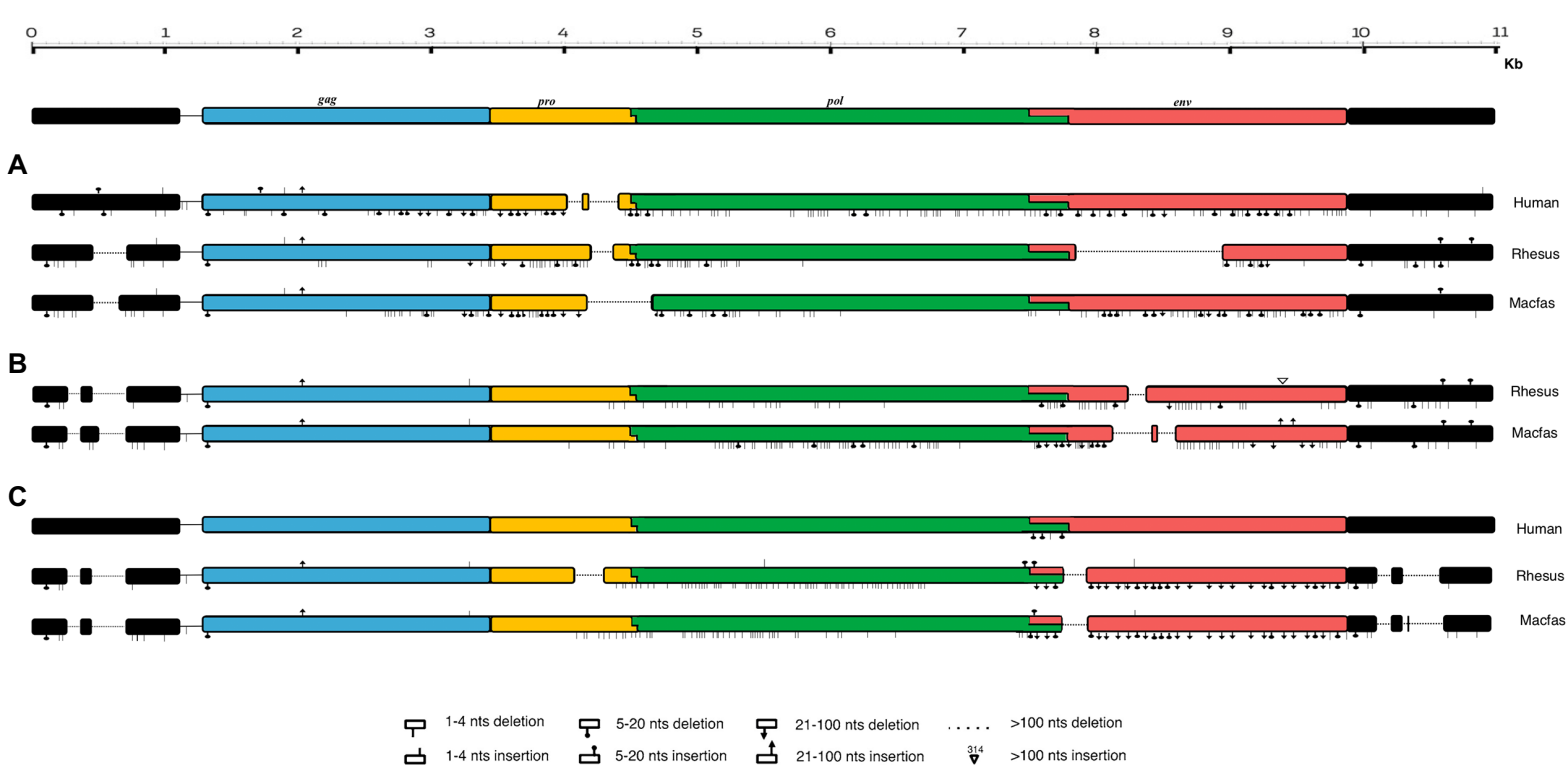
Schematic representation of HML2 proviruses in Human and Macaque genomes. Representative Consensus of HML2 proviruses **(A)** Shared between human and macaques. **(B)** Macaque-shared HML2 sequences. **(C)** Species-Specific Sequences.

For structural characterization, typical HML2 prototype features present in the Dfam database were used, which includes the internal coding region for *gag* (~156–2,297), *pro* (~2063–3,082), *pol* (~3,037–5,785), and *env* (~5,624–7,951). Considering the full-length human HML2 proviral structure, we analyzed in detail the nucleotide structure of all the full-length HML2 sequences in macfas and rhesus separately to gain a better understanding of the complete proviral structure in macaques’ genomes. For this, using the complete alignment of macfas and rhesus, we assessed that the mean length of HML2 proviral sequences was 6,158 and 7,143 bp, respectively, with lower length as compared to that of human HML2 (7,536 bp) due to the presence of several deletions, considering the Dfam reference that affects a subset of the retrieved elements. In these more defective proviruses, the deletions removed parts of the 3′ half of the HML2 genome from the terminal portion of *pro* to the *env* gene. The overall GC content of HML2 elements for both Macaca species was 40%–41%, with a comparable enrichment in AT nucleotides in the human HML2 reference sequence (32.5% and 25.2%, respectively), macfas (31.8% and 26.6%, respectively), and rhesus (31.9% and 26.4%, respectively). To characterize the structural completeness of the individual genes of HML2 elements, we annotated their sequences as per the Dfam HML2 prototype features. An inferred structure of HML2 provirus has been schematically represented in Figure 4, which depicts the consensus HML2 sequences of the three categories, i.e., human-shared HML2 proviruses; macaque-shared HML2 sequences, and Species Specific HML2.

**Figure 5:**
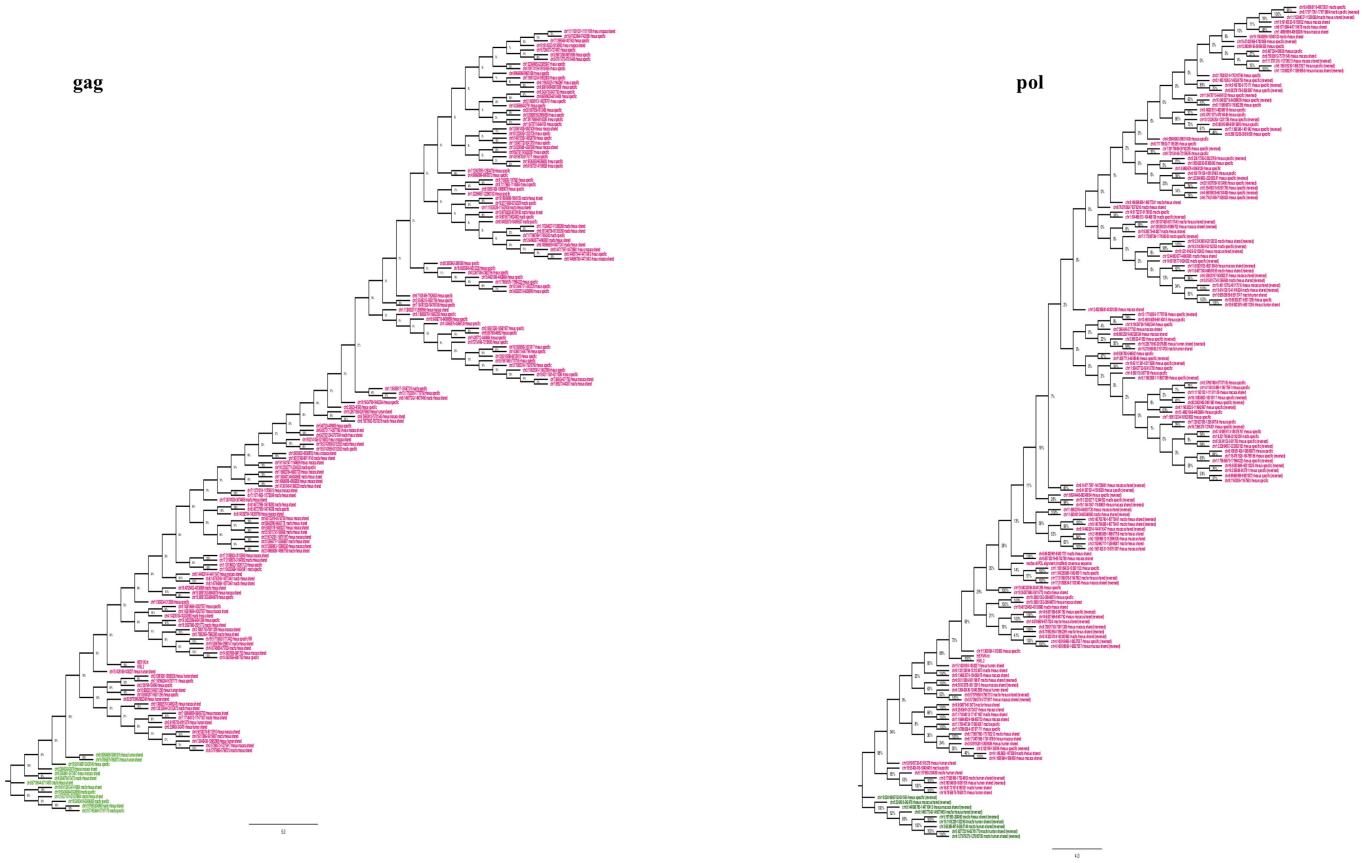
Phylogeny of HML2 gag and pol genes of macaque species. The trees represent the conservative nature of *gag* and *pol* genes of HML2 in both the Macaca species with the major upper cluster of bootstraps 100% (*gag*) and 98% (*pol*). The major cluster is highlighted in pink while the ones highlighted in green are the outliers.

**Figure 6:**
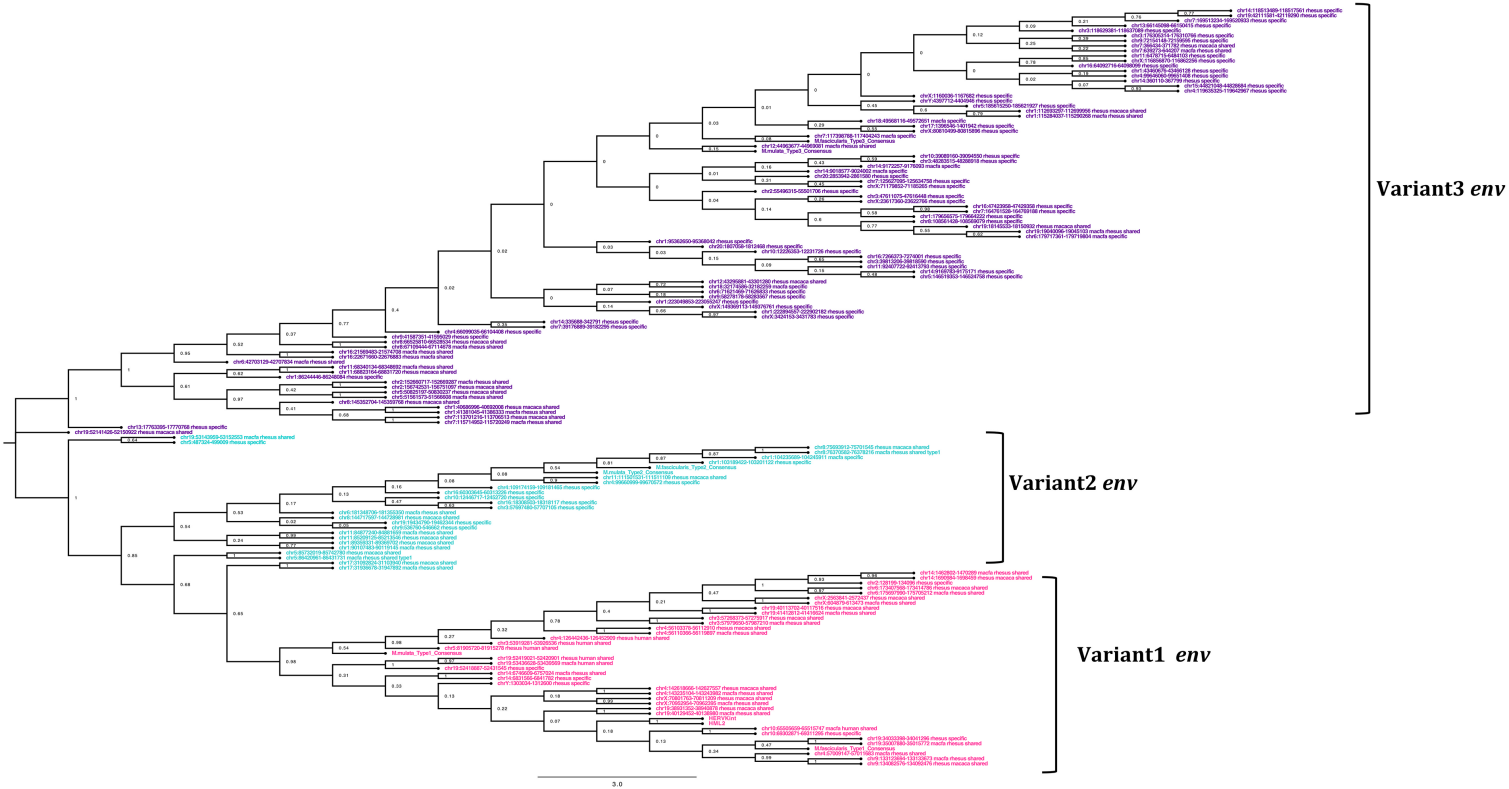
Phylogeny of the *env* gene of HML2 in *Macaca fascicularis* and *Macaca mulatta*. The clusters indicate the presence of 3 subtypes of *env* in Old World Monkeys. The type1 (pink) is similar to the human HML2 type2 (rec) *env*, while type 2 (cyan) has specific insertions in the transmembrane region, and type 3 (purple) which forms the largest cluster mostly species-specific and some shared between the two macaques having HERVK11 insertion.

In general, the nucleotide structure analysis revealed the mean length for *gag, pol,* and *env* genes to be 1,899, 1,908, and 1,851 bp for macfas, respectively, and 1,898, 1,803, and 2,312 bp for rhesus, respectively. We hence aligned the *gag, pol,* and *env* genes of macfas and rhesus to the reference HML2 genes, to assess the conservative as well as the divergent nature of the genes. To better understand the variability in *gag, pol,* and *env* genes within the Macaca species, we generated NJ trees for all three genes. As presented in Figures 5A,B, major clustering was observed for *gag* and *pol* genes, with most of the sequences being included in a main cluster with a bootstrap of 100% and 98%, respectively, indicating a highly conserved nature of both the genes in HML2 sequences of Macaca species. Only a few elements were grouped separately in both *gag* and *pol* trees, forming a minor cluster with 80% and 100% of bootstrap support, respectively (Figure 5). Differently, the *env* genes showed a very divergent nature based on the phylogeny, and two major clusters with 100% bootstrap support were observed in *env* NJ tree (Figure 6). In these clusters, sequences have been classified according to the *env* gene structure into 3 variants, as discussed below about the divergent nature of this gene. Such variants were also supported by bootstrap values in the corresponding phylogenetic clusters. The upper major cluster (violet) included 79 *env* sequences, while the lower cluster was formed by 60 *env* sequences, including 35 of them in a highly supported subcluster (pink, bootstrap = 98%). From the alignments performed and the phylogenetic analyses for the three genes in Macaca species, we observed a remarkable divergence among macaques’ HML2 *gag* and *env* genes due to specific insertions and deletions in the different clusters, while the *pol* gene was conserved in the HML2 elements of the macaques. For this reason, we further focused on identifying the main differences in macaques *gag* and *env* structure as compared to human HML2 genes.

#### Divergent nature of *gag* gene

**Figure 7:**
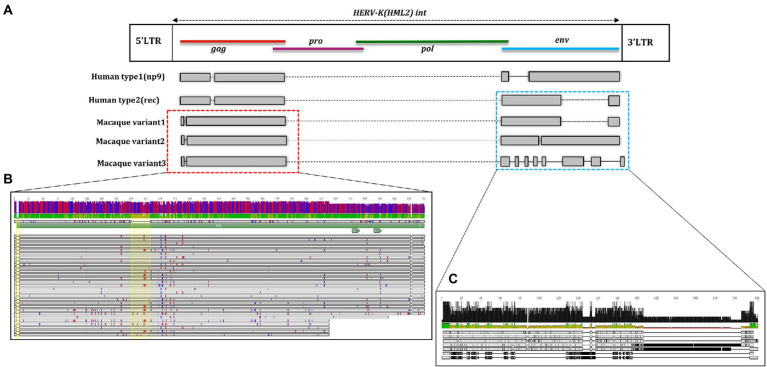
Structural representation of *gag* and *env* genes HML2 with respect to the consensus generated from potential residual ORFs of Human and Macaque species (*Macaca fascicularis* and *Macaca mulatta*). **(A)** The variants 1, 2, and 3 are specified based on the variation in the env region. The solid black lines refer to the deletion in the regions while the dotted lines are the stretch based on the insertions in that specific region. **(B)** Alignment of potential coding capacity of the *gag* genes of both the macaque species (described in detail in Table 3). Characteristics indels in macaque gag proteins as compared to human ones are highlighted with yellow blocks. **(C)** Alignment of the consensus generated for each cluster in both *M. fascicularis* (Macfas) and *M. mulatta* (Rhesus), the alignment of the three variants in both species are according to that indicates in **(A)**.

The alignment and tree of the *gag* region showed that its genic portion was quite conserved among HML2 macaque elements and was also ~70% similar to the human HML2 *gag* region (data not shown). However, in the *gag* gene, we identified some characteristic deletions and insertions present in all the macaque sequences (Figure 7A). Particularly, we observed a recurrent deletion of 12 bp (corresponding to nucleotides 156–167 of the reference) that was found exclusively in macaques HML2 and not in human sequences. In addition, a 96 bp insertion (found between nucleotides 745–838 of the reference) is stably found in the macaque sequences, being represented in some of human loci (27%). Besides structural integrity, we translated the identified *gag* sequences to gain insights on their residual coding potential in terms of stop codons and frameshift mutations. We hence identified 9 and 20 potentially coding *gag* ORFs without any stop codon or frameshift in macfas and rhesus, respectively (Figure 7B; Table 3). The HERV *gag* genes consist of CA, MA, and NC functional domains, and include in HERV-K groups two zinc finger motifs (CX_2_CX_4_HX_4_C; Jern et al., 2005) that were correctly identified in macaque HML2 *gag* genes as well by the NCBI Conserved Domains and UniProt annotations (Table 3). Out of the above 29 potentially coding *gag* ORFs, 5 *gag* ORFs of rhesus were shorter in length, missing the 3′ portion that include the two zinc finger motifs and retaining only the CA and MA functional domains. Translated Gag proteins had a length of 701 aa while the 5 truncated ORFs coded for shorter proteins of 538 aa (Table 3).

**Table 3:** Presence of structural and functional domains in the potential *gag* ORFs.

Name	Amino acid length (aa)	CA	MA	NC	Zn Finger motifs (CX_2_CX_4_HX_4_C)
*Macaca fascicularis* **(macfas)**					
chr12:95783035–95,789,182	701	✓	✓	✓	✓
chr14:9018577–9,024,002	701	✓	✓	✓	✓
chr19:19040096–19,045,103	701	✓	✓	✓	✓
chr1:115284037–115,290,268	701	✓	✓	✓	✓
chr7:117398788–117,404,243	701	✓	✓	✓	✓
chr7:639273–644,207	701	✓	✓	✓	✓
chr6:42703129–42,707,834	701	✓	✓	✓	✓
chr8:76370582–76,378,216	701	✓	✓	✓	✓
chr8:104092873–104,095,507	701	✓	✓	✓	✓
*Macaca mulatta* **(rhesus)**					
chrX:1160036–1,167,682	701	✓	✓	✓	✓
chrX:71179852–71,185,265	701	✓	✓	✓	✓
chr7:39176889–39,182,295	701	✓	✓	✓	✓
chrX:3424153–3,431,783	701	✓	✓	✓	✓
chr3:118629381–118,637,089	701	✓	✓	✓	✓
chrX:149369113–149,376,761	701	✓	✓	✓	✓
chr4:99646060–99,651,408	701	✓	✓	✓	✓
chr16:60303645–60,313,226	701	✓	✓	✓	✓
chr1:95362650–95,368,042	701	✓	✓	✓	✓
chr5:146519353–146,524,758	701	✓	✓	✓	✓
chrX:23617360–23,622,766	701	✓	✓	✓	✓
chr3:57697480–57,707,105	701	✓	✓	✓	✓
chr13:66145098–66,150,415	701	✓	✓	✓	✓
chrX:116856870–116,862,256	701	✓	✓	✓	✓
chr16:64092716–64,098,099	701	✓	✓	✓	✓
chr8:104092873–104,095,507	639	✓	✓	✓	✓
chr6:42073171–42,077,892	538	✓	✓	–	–
chr8:75693912–75,701,545	538	✓	✓	–	–
chr11:111501531–111,511,109	538	✓	✓	–	–
chr7:366434–371,782	538	✓	✓	–	–
chr1:112693297–112,699,956	538	✓	✓	–	–

✓ indicates the presence of the gag portions; − indicates that the portions are absent. CA, capsid; MA, matrix; NC, nucleocapsid.

#### Divergent nature of *pol* gene

The genic portion of the *pol* region of the macaque HML2 elements was quite conserved, showing a ~ 80% identity with respect to Dfam reference (data not shown). Similar to that of the *gag* gene, this similarity was confirmed by performing sequence alignment and phylogenetic analysis with the human reference *pol* gene. No insertions or deletions were seen in macaque HML2. To gain insights on the residual coding capacity of the *pol* gene, we translated its sequences and identified 7 potentially coding ORFs, i.e., 6 in rhesus and 1 in macfas, without any stop codon or frameshift mutations (Table 4). Finally, all the functional domains present in the *pol* gene (RT, RH, and IN) were correctly identified in the above macaque HML2 *pol* regions using NCBI conserved domain and UniProt. The translated Pol proteins were 881 aa in length.

**Table 4:** Presence of structural and functional domains in the potential *pol* ORFs.

Name	Amino acid length (aa)	RT	RH	IN
*Macaca fascicularis* **(macfas)**				
chr18:32174586–32,182,259	881	✓	✓	✓
*Macaca mulatta* **(rhesus)**				
chr3:118629381–118,637,089	881	✓	✓	✓
chr4:119635325–119,642,967	881	✓	✓	✓
chr19:42111581–42,119,290	881	✓	✓	✓
chr20:2853942–2,861,580	881	✓	✓	✓
chrX:1160036–1,167,682	881	✓	✓	✓
chrX:3424153–3,431,783	881	✓	✓	✓

✓ indicates presence of pol portions. RT, Reverse Transcriptase; RH, RNAse H; IN, Integrase.

#### Divergent nature of *env* gene

Differently from the *gag* gene, the *env* alignment of both the macaque sequences showed a wide divergence among sequences and with respect to human HML2. As previously mentioned, human HML2 shows 2 subtypes based on the divergence in the env region. The type 1 HML2 has a 292 bp deletion leading to the production of Np9, while type 2 lacks this deletion and hence, it produces the alternative protein Rec (Acton et al., 2019; Xue et al., 2020). To characterize the presence of type II (rec) HML2 env in OWMs in more detail, we annotated the Rec portions of the reference sequence and performed the alignment with all the *env* genes of the macaque species. We analyzed the presence of complete Rec portions in 58 env genes of both species encoding for the putative Rec protein. We further evaluated the presence of two important functional domains for the Rec proteins, i.e., the nuclear localization signal (NLS) motif and the nuclear export signal (NES) motifs. Analysis revealed that these motifs were in fact present in all the 58 *env* genes. Hence, we confirmed the presence of type II (rec) HML2 *env* in the macaque species and so we further focused on analyzing the structural integrity and completeness of *env* gene.

**Figure 8:**
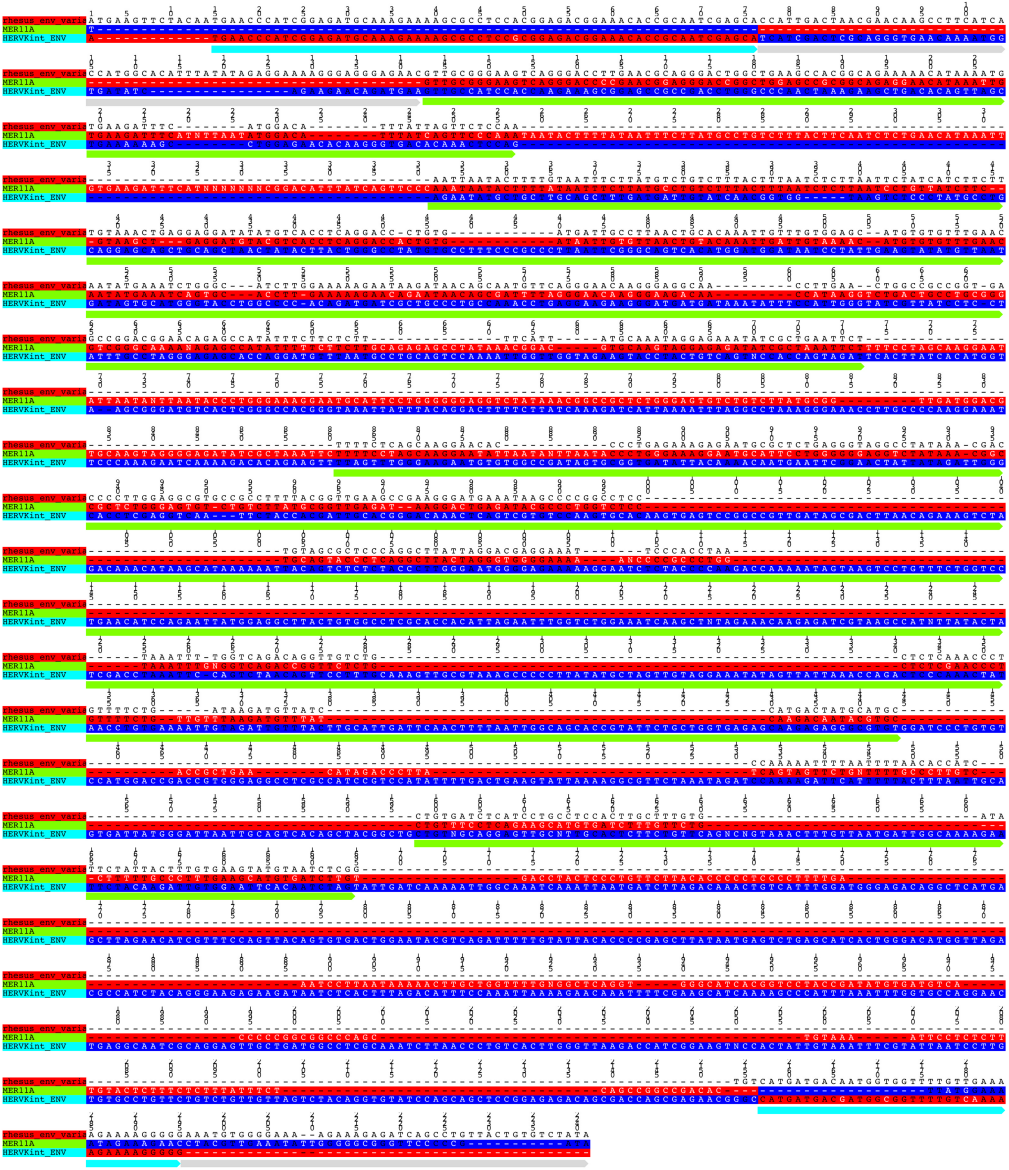
Test of recombination between macaque HML2 env variant3 and HML8 MER11A LTR The portions deriving from HML2 *env* gene (cyan) and MER11A LTR (green) are annotated along rhesus consensus for *env* variant3.

From the alignment of all the *env* genes of Macaca species and the clusters observed in the *env* phylogenetic tree (Figure 6), we categorized these sequences based on the presence of recurrent insertions and deletions at a specific position with respect to reference *env*, and designated them as variant 1, variant 2, and variant 3 (Figures 6, 7C). The variant1 *env* was phylogenetically related to variant2, forming a specific subcluster of 35 sequences that shared similarities with the reference HML2 *env* in terms of structural completeness. Accordingly, the consensus generated from the variant1 *env* of macaques had ~80% similarity with the human reference gene, also showing the potential to encode for a complete Env protein (Figure 7C), while neither of the individual *env* sequences of this variant nor the other variants encode for a potential ORF, as all of them had stop codons and frameshift mutations. The remaining 23 sequences were classified as variant2 and had a characteristic 191 bp insertion in the transmembrane region (Figures 6, 7C). Except for this insertion, variant2 was also rather similar to the human HML2 *env* genes. The third and bigger cluster was instead observed to be very divergent from the other two clusters and included 81 sequences (Figures 6, 7C). Specifically, most members of such cluster were rhesus-specific and macfas-specific HML2, plus very few elements shared between the two macaques (Figure 6). This cluster had several recurrent deletions and was hence the most divergent from the reference human HML2 *env* region (Figure 7C). For this reason, we decided to further study its structure, and we identified the presence of a common HERVK11/HML8 insertion with the stretch of 673 nucleotides that was found in all the variant3 *env* macaque sequences. Of note, such integration was found in macaque HML2 elements only, being completely absent from human HML2 loci. The HML8 insertion in the Repeat masker annotations is reported as MER11A, being one of the three LTR types associated to the group, and is stably found in transmembrane region (TM) of the variant3 HML2 *env* gene, suggesting some ancestral recombination events between HML2 *env* and HML8 LTRs specific to macaques. To test such hypothesis, we analyzed rhesus *env* variant3 consensus sequence along with HML2 *env* gene and MER11A HML8 LTR through the software Recco (Figure 8). Results confirmed that most of the *env* of macaque variant3 sequences has been replaced by the recurrent integration of HML8 LTR MER11A, leaving only short portions of the original 5′ and 3′ ends of the gene (Figure 8).

## Discussion

The HML2 integrations in the human genome are highly studied for their possible roles in various normal and diseased conditions, differently from that of the non-human primates that are still poorly characterized. The integration and evolution in the non-human primates over millions of years remains unclear and hence, the study provides a detailed analysis of HML2 proviruses in Macaque genomes in terms of integrations, evolution, and structural variations.

To do this, the first step was to retrieve all HML2 elements in the available OWM genome assemblies, with a particular attention to macaques due to their relevance as disease models. HML2 sequences were collected by a double approach based on comparative genomics and *de novo* identification. In this way, we retrieved all members of the HML2 clade in OWMs, namely Green Monkey (81), Macfas (77), Rhesus (131), Baboon (76), and Proboscis Monkey (102; Table 1). Even though we identified HML2 integrations in all the available genome assemblies of OWM and characterized the ones shared with humans (Supplementary Figure 1), further comparison with other non-human primates was difficult due to poor comparative genomics annotations among OWM assemblies. Therefore, we focused on *Macaca fascicularis* (macfas) and *Macaca mulatta* (rhesus), as these are the most used non-human primates for the biomedical research (Bunlungsup et al., 2017). Combining the two approaches mentioned earlier, we identified a total of 208 HML2 integrations in the macaque genomes, i.e., the rest were specific to either macfas (Salavatiha et al., 2020) or rhesus (73; Table 2). As studies suggest that rhesus and macfas form hybrid species, the possibility of 46 macaque-shared HML2 integrations can also be due to the relatively recent hybridization events between the two species (Kanthaswamy et al., 2009; Bunlungsup et al., 2017). A complete dataset of the HML2 integrations in humans, *M. fascicularis,* and *M. mulatta* identified in the study is provided in supplementary tables. In literature, a single study was focused on identifying the presence of HML2 in one of the considered primates, namely Rhesus macaques, reporting 19 HML2 proviruses designated as RhERV-K (Romano et al., 2007). However, direct comparison with such a result was not possible due to the absence of nucleotide sequences and genomic coordinates for these integrations. According to the studies performed by Belshaw et al. (2005) and Marchi et al. (2014), we know that due to the recent activity of HML2 in humans, HML2 group includes several polymorphic integrations, i.e., integrations that are present in only a portion of human population, and are hence not represented in the human genome assembly (Belshaw et al., 2005; Marchi et al., 2014). Considering a similar prolonged activity of HML2 in macaques, a comparable situation of polymorphic insertions might be possible. Hence, further studies on individual macaque genomes from different populations are needed to evaluate the presence of such polymorphic integrations in detail.

Using the data obtained from both the approaches, we carried out phylogenetic analysis and age estimation studies to understand the evolutionary patterns of HML2 group in both the macaques. The phylogenetic analysis confirmed that the HML2 elements found in human genome are divergent from the OWM ones, forming a separate cluster (Figure 1). Similarly, separate clustering was observed for the minority of HML2 sequences of macaques shared with humans, as well as for those shared between macfas and rhesus and the HML2 sequences specific to either of the macaque species (Figure 2). The snapshot about HML2 dynamics of acquisition provided with the present study is in line with the previous studies about HML2 in humans, confirming that the integration of HML2 in the primate germline occurred only in *Catarrhini* parvorder (including *Hominidae* and OWMs), being hence absent in *Platyrrhini* (also called New World Monkeys) that diverged from *Catarrhini* around 43 mya (Mayer et al., 1998; Bannert and Kurth, 2006). In addition, we found that only a minority of HML2 loci found in macaques is shared with humans, deriving hence from the infection of OWM and *Hominidae* common ancestor by a “primordial” circulating HML2. On the contrary, the fact that most macaque HML2 sequences are not shared with humans indicates that most of them were acquired after the evolutionary split between macaques and Hominidae, i.e., less than 30 mya. This observation, along with the abundance of macaque-specific integrations, further confirms that the group had a prolonged diffusion in primates, leading to several integrations shared only by macaques or even specific to a single Macaca species.

To gain more insights on the diffusion of HML2 in macaques, we estimated each sequence time of integration by combining the traditional method based on nucleotide divergence between the LTRs of the same proviruses with the comparison of individual LTRs as well as *gag* and *pol* genes with respect to a representative consensus sequence. Results confirmed that the diffusion of HML2 loci in primates initiated around 43–30 mya (Figure 3), sharing a common ancestral activity of proviral HML2. The major wave of HML2 acquisition accounting for the macaque-specific integrations occurred between 30 and 7 mya, with the latest tail of species-specific integrations after the two macaques evolutionary split, i.e., less than 2.5–5 mya. This estimation, along with the other results obtained in the present study, is in line with what was observed in a study by Romano et al. (2007), limited to 19 HML2 insertions in rhesus genome, in which 47% of such elements had estimated integration times lower than 4.5 million years (Romano et al., 2007). However, the considered 19 HML2 insertions were probably among the youngest one, being likely macaque-specific, given that the reported average age for them (10.3 mya) is much lower as compared to the wide range of acquisition of HML2 elements by macaques when considering the whole set of integrations, as done in the present study. Their specificity to rhesus is also suggested by the fact that they formed a distinct phylogenetic cluster when compared to a set of human and chimpanzee HML2 (Romano et al., 2007). Overall, the results obtained about HML2 colonization in macaque genomes reveal that, similarly to the prolonged diffusion reported in humans, the group had a prolonged parallel distribution in OWMs that started at the beginning of *Catarrhini* speciation and provided new integrations until very recently.

To further evaluate the hypothesis of divergent nature of macaque HML2 proviruses with respect to human elements, we analyzed the phylogeny (Figures 5, 6) and structural features (Figure 7) of full-length HML2 proviruses focusing on *gag*, *pol,* and *env* genes. The *gag* and *pol* genes in the two macaque species are well conserved which differs from human HML2 one for having a specific insertion (96 bp) and a recurrent deletion (12 bp) in the *gag* gene, while no such event being observed in the *pol* gene. We assessed that 29 *gag* genes and 7 *pol* genes retain ORFs with potential coding capacity for polyproteins of 538–701 aa for *gag* and 881 aa for *pol* (Tables 3, 4). As previously mentioned, the *env* gene in human HML2 has two subtypes: type I (Np9) and type II (Rec) based on the presence or absence of a 292 bp deletion, respectively (Subramanian et al., 2011). Based on what reported in Heyne et al. (2015), the mutational event that led to the emergence of the type I HML2 occurred after the split between *Hominidae* and OWMs, being hence absent in the latter. Accordingly, we confirmed the presence of only type II (Rec) *env* by identifying the putative *rec* coding region in 58 *env* sequences of both macaque species, also retaining the two important Rec motifs, i.e., NLS and NES. These 58 *env* sequences include *env* variants 1 and 2 only, while the rest of the *env* genes devoid of the rec domain were mostly belonging to variant 3, as expected due to the supposed recombination event between the HML2 env gene and MER11A LTR of HML8. In this regard, the above hypothesis was tested using Recco software, confirming that most of the variant3 *env* (including the eventual Rec domain) has been replaced by recombination with HML8 LTR MER11A, leaving only short portions of the original 5′ and 3′ ends of the gene. This recombination event is likely occurred during the process of endogenization, leading to the intragenomic emergence of a separate form of HML2 subtype in macaques, since this specific insertion has not been observed in the human HML2 loci. Furthermore, this insertion was found in the macfas- and rhesus-specific *env* genes (and very few macaque-shared genes) suggesting that the above recombination activity occurred recently in macaques, right before the split between macfas and rhesus species. This is in line also with the prevalence of such variant, as emerged during the major period of activity of the group in OWMs (Figure 3). However, all the three variants of HML2 were likely circulating at the same time in OWMs, given that they all include HML2 integrations specific to the individual Macaca species. Similar events of recombination have been previously reported in Vargiu et al. (2016) leading to the formation of mosaic forms of HERVs, among which the HML1, HML2, and HML3 HERVK groups are most frequently involved (Vargiu et al., 2016). Noteworthy, such recombination events often involved the *env* gene, in a phenomenon termed as “*env* snatching” that can further favor the amplification of such variants by the loss of the envelope and the subsequent enhancement of intragenomic spread instead of active infection (Vargiu et al., 2016). Hence, while *env* snatching is a widespread phenomenon among the retroviral integrations in the human lineage, it has not been previously reported in OWMs HERVs, thus representing a novel feature of HML2 group in these primates.

## Conclusion

The overall characterization of HML2 integrations in *M. fascicularis* and *M. mulatta* provides the first exhaustive genomic description of HML2 in OWMs in terms of evolutionary endogenization, structure, and coding capacity.

Phylogeny and time of acquisition estimates confirmed a prolonged activity of the HML2 group in macaques, accounting for human-shared, macaque shared, and species-specific integrations. If on the one side the different time of acquisition for these 3 subsets of sequences surely accounts for diverse accumulation of nucleotide divergence, on the other side the diversity observed among them and the common features of HML2 sequences acquired in the same period likely suggest the ancestral circulation of different exogenous variants, possibly with specific tropism for the individual macaque species in the case of species-specific integrations.

The study also reports for the first time the presence of three viral variants in macaques, based on the presence of alternative *env* forms. One of these seems specific to macaques, and likely arose from the recombination with a HML8 LTR. Overall, these results provide a deeper understanding about the dynamics of diffusion of the group in primates, serving as a starting point to study the ancestral tropism of HML2 in human as well as non-human primates.

## Data availability statement

The original contributions presented in the study are included in the article/Supplementary material, further inquiries can be directed to the corresponding author.

## Author contributions

SC performed the analysis and wrote the manuscript. NG supervised the analyses, checked the data, and participated in editing the manuscript. ET conceived and coordinated the study. All authors contributed to the article and approved the submitted version.

## Conflict of interest

The authors declare that the research was conducted in the absence of any commercial or financial relationships that could be construed as a potential conflict of interest.

## Publisher’s note

All claims expressed in this article are solely those of the authors and do not necessarily represent those of their affiliated organizations, or those of the publisher, the editors and the reviewers. Any product that may be evaluated in this article, or claim that may be made by its manufacturer, is not guaranteed or endorsed by the publisher.

## Supplementary material

SUPPLEMENTARY FIGURE S1 Phylogeny of the HML2 proviruses shared between Human and Non-Human Primates. Neighbor Joining tree was generated for full-length proviruses of Green Monkey (orange), Rhesus (blue), Macfas (green), Baboon (cyan) and Proboscis Monkey (pink) and human HML2 (red).

SUPPLEMENTARY TABLE S1 Human HML2 coordinates.

SUPPLEMENTARY TABLE S2 Macaque shared HML2 coordinates.

SUPPLEMENTARY TABLE S3 *Macaca fascicularis* HML2 coordinates.

SUPPLEMENTARY TABLE S4 *Macaca mulatta* HML2 coordinates.
